# Volvulus de la vésicule biliaire: à propos d'une observation

**DOI:** 10.11604/pamj.2015.20.228.6294

**Published:** 2015-03-12

**Authors:** Foulaké Tandian, Mamadou Cissé, Alpha Oumar Touré, Mamadou Seck, Ousmane Thiam, Mohamadou Lamine Guèye

**Affiliations:** 1 Service de Chirurgie Générale, CHU Aristide Le Dantec, Dakar, Sénégal

**Keywords:** Volvulus, vésicule biliaire, cholécystectomie, Volvulus, gallbladder, cholecystectomy

## Abstract

Le volvulus de la vésicule biliaire (VVB) est une affection rare et de diagnostic difficile; le plus souvent per opératoire. Nous rapportons 1 cas de VVB avec gangrène de la vésicule biliaire chez un enfant de 10 ans. Le diagnostic a été porté à l'exploration chirurgicale réalisée en urgence pour un syndrome occlusif fébrile. Il s'agissait d'un volvulus total de la vésicule biliaire. A cette occasion, nous rappelons les aspects diagnostiques et thérapeutiques de cette affection.

## Introduction

Le volvulus de la vésicule biliaire est une rotation de la vésicule autour de son pédicule cystique. Elle survient sur une vésicule ayant une disposition anatomique particulière. Le diagnostic peut être porté à l’échographie. Mais dans la majorité des cas, il s'agit d'un diagnostic per-opératoire. Nous rapportons 1 cas particulier par son mode de révélation et son diagnostic tardif au stade de gangrène.

## Patient et observation

Il s'agissait d'un enfant âgé de 10 ans, de sexe masculin, sans antécédents pathologiques particuliers, adressé par un centre de santé pour la prise en charge de douleurs abdominales aiguës. Le tableau évoluait depuis 48 heures, marqué par des douleurs de la région ombilicale et de l'hypochondre droit, intenses et sans irradiation particulière. Ces douleurs étaient associées à des vomissements bilieux et à un arrêt des matières et des gaz depuis 24 heures. L'examen physique retrouvait un enfant hypotonique, un faciès terreux, des sueurs profuses avec une fièvre à 39°C, une tachycardie à 112 batts/min, une polypnée à 42 cycles/min et une tension artérielle de 90/60 mmHg. La palpation retrouvait une sensibilité abdominale, une défense de la région ombilicale et de l'hypochondre droit. La numération formule sanguine notait une hyperleucocytose à 15 000 éléments/mm^3^ avec polynucléose. Le taux d'hémoglobine était de 10,7g/dl et les plaquettes à 23000. La radiographie de l'abdomen sans préparation montrait une anse sentinelle au niveau du flanc droit.

**Figure 1:**
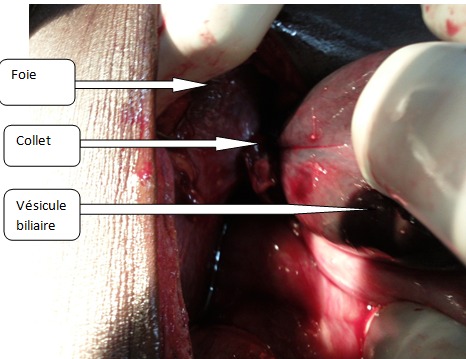
Aspect per opératoire du volvulus de la vésicule biliaire

**Figure 2:**
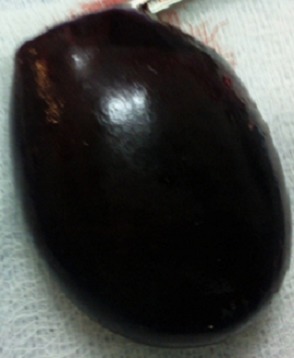
Aspect de la vésicule après ablation

Une laparotomie a été réalisée en urgence après une réanimation pré opératoire. L'exploration a retrouvé une rotation de la vésicule biliaire autour du canal cystique à un tour de spire dans le sens antihoraire. La Figure 1 montre l'aspect du volvulus à l'exploration. La vésicule était gangrenée, ptosée, distendue et désinserrée du foie. Le mésocholécyste était très mince. Il existait un petit épanchement péri vésiculaire et un magma adhérentiel avec l’épiploon. Nous avons procédé à une détorsion et à une cholécystectomie antérograde. Une toilette abdominale et une fermeture sans drainage ont été effectuées. A l'ouverture de la pièce opératoire, la paroi de la vésicule était épaissie et il n'y avait pas de calcul. La Figure 2 montre l'aspect gangréné de la vésicule après ablation. Les suites étaient simples. Le patient est sorti au 5ème jour post opératoire. Il a été revu au 14ème puis au 30ème jour sans aucune plainte. L'examen anatomopathologique a confirmé une cholécystite aiguë alithiasique.

## Discussion

**Epidémiologie:** L’âge de survenu du VVB se situe après 60 ans dans la littérature [1]. Aucun facteur n'est retrouvé pour justifier cette fréquence chez les personnes âgées. Notre observation fait partie des rares cas rapportés chez l'enfant dont le plus jeune cas rapporté a 2 ans [2]. Il existe une prédilection chez la femme du volvulus de la vésicule, soit 3 femmes pour 1 homme [2]. Ces données épidémiologiques sont difficiles à analyser vu le petit nombre de cas rapporté par étude. **GROSS** a décrit des dispositions anatomiques favorisant le **VVB** classées en 3 types [3]: **Type I:** vésicule biliaire pédiculée, amarrée dans son propre péritoine, libre dans l'abdomen, réuni au pédicule biliaire par le mésocyste qui peut se tordre; **Type II:** la vésicule et le canal cystique sont amarrés à face inférieure du foie par le mésocyste. La torsion de ces 2 type donnent le volvulus total; **Type III:** La vésicule en sablier due à un développement incomplet est à l'origine du volvulus partiel avec torsion à la jonction de la partie adhérente et libre [3]. Les anomalies retrouvées chez notre patient correspondent au type I de GROSS. Selon **Descottes**, le volvulus de la vésicule survient chez les personnes maigres, présentant une cyphoscoliose [4]. Cependant, certains auteurs n'ont pas retrouvé de tare [1]. Aucune morphologie particulière n'a été notée chez notre patient. Par ailleurs, des facteurs déclenchant le volvulus de la vésicule biliaire ont été rapportés dans certaines études notamment: repas trop copieux; effort violent; traumatisme; grossesse ou accouchement [1]. Nous n'avons pas retrouvé une cause déclenchante chez notre patient.

**Diagnostic:** Les signes fonctionnels ne sont pas spécifiques et sont dominés par un syndrome douloureux abdominal siégeant à l′épigastre, au flanc droit comme dans notre cas ou à la fosse iliaque droite [1]. L’état général est généralement conservé. Les signes de gravité sont rencontrés dans les formes compliquées de cholépéritoine ou de gangrène comme chez notre patient [1]. L'examen physique peut retrouver une défense de l′hypochondre droit ou de tout l'hémi abdomen droit [1]. La palpation de la vésicule volvulée, sous le rebord costal droit et qui réalise le signe de “va et vient“ vésiculaire de **SHORT** est pathognomonique [5]. Ce signe est difficile à retrouver à cause du météorisme abdominal et de la défense [1]. Nous ne l'avons pas mis en évidence chez notre patient. A la biologie, il existe fréquemment une hyperleucocytose avec polynucléose [1]. La radiographie de l'abdomen sans préparation est peu contributive. Elle peut montrer une anse sentinelle, faisant penser à une appendicite surtout lorsqu'elle est associée à une polynucléose [4]. C’était le cas chez notre patient. L’échographie abdominale permet de montrer une vésicule biliaire tendue, avec une paroi épaissie et alithiasique. Nous ne l'avons pas réalisée chez notre patient. Ces examens permettent rarement de faire le diagnostic pré opératoire. L'intervention est le plus souvent réalisée pour suspicion d'une autre pathologie (appendicite aiguë, cholécystite) comme chez notre patient. Le scanner et l'IRM sont rarement pratiqués en situation d'urgence dans notre contexte. Ils permettent un diagnostic plus précis que l’échographie.

**Traitement:** Le traitement du VVB est exclusivement chirurgical. Il consiste à réaliser une cholécystectomie [6]. L'exploration permet de rechercher les facteurs favorisants. La cholécystectomie doit être réalisée sans détorsion pour certains auteurs pour éviter l'ensemencement microbien de la voix biliaire principale [7]. Cependant d'autres recommandent une détorsion première pour éviter une lésion de cette voix biliaire principale [1]. C'est ce qui a été réalisé chez notre patient. Nous n'avons pas observé d'infections des voix biliaires en post opératoire.

## Conclusion

Le **VVB** est une affection rare surtout décrit chez la personne âgée. Cette observation est une contribution sur la possibilité de survenue de cette maladie chez l'enfant sans morphologie particulière ni facteur déclenchant. Il s'agit d'une urgence chirurgicale qui nécessite une cholécystectomie en urgence pour éviter la survenue de la gangrène de la vésicule biliaire et ses complications.
